# Risk for progression to type 1 diabetes in first-degree relatives under 50 years of age

**DOI:** 10.3389/fendo.2024.1411686

**Published:** 2024-08-12

**Authors:** Ines Urrutia, Rosa Martinez, Begona Calvo, Irene Marcelo, Laura Saso-Jimenez, Idoia Martinez de Lapiscina, Jose Ramon Bilbao, Luis Castano, Itxaso Rica, The Collaborative Working Group, A. Aguayo

**Affiliations:** ^1^ Biobizkaia Health Research Institute, Barakaldo, Spain; ^2^ UPV/EHU, CIBERDEM, CIBERER, Endo-ERN, Barakaldo, Spain; ^3^ Department of Medical Oncology, Cruces University Hospital, Barakaldo, Spain; ^4^ Hospital de Mataró - Consorci Sanitari del Maresme, Barcelona, Spain; ^5^ Pediatric Endocrinology Unit, Cruces University Hospital, Barakaldo, Spain

**Keywords:** type 1 diabetes, first-degree relatives, pancreatic autoantibodies, autoimmunity, prediction, risk

## Abstract

**Introduction:**

The detection of pancreatic autoantibodies in first-degree relatives of patients with type 1 diabetes (T1D) is considered a risk factor for disease. Novel available immunotherapies to delay T1D progression highlight the importance of identifying individuals at risk who might benefit from emerging treatments. The objective was to assess the autoimmunity in first-degree relatives of patients with T1D, estimate the time from autoimmunity detection to the onset of clinical diabetes, and identify the associated risk factors.

**Methods:**

Retrospective multicenter study of 3,015 first-degree relatives of patients with T1D recruited between 1992 and 2018. Pancreatic autoantibodies (IAA, GADA, IA2A, and ZnT8A) were determined by radioimmunoassay, starting the analyses at diagnosis of the proband. All those with positive autoimmunity and normal fasting blood glucose without clinical symptoms of diabetes were followed up in the study. The progression rate to T1D was assessed according to sex, relationship with the proband, age at autoimmunity detection, type/number of autoantibodies, and HLA-DRB1 genotype. Cox proportional-hazard models and Kaplan–Meier survival plots were used for statistical analyses.

**Results:**

Among the relatives, 21 progenitors [43.7 years (IQR: 38.1–47.7)] and 27 siblings [7.6 years (IQR: 5.8–16.1)] had positive autoantibodies. Of these, 54.2% (95% CI: 39.2%–68.6%) developed T1D (age at autoimmunity detection 11 months to 39 years) in a median of 5 years (IQR: 3.6–8.7; ranged from 0.9 to 22.6 years). Risk factors associated with faster progression to T1D were multiple autoimmunity and <20 years at autoimmunity detection. Younger relatives (<20 years) with multiple autoantibodies had a 5-year cumulative risk of developing diabetes of 52.9% (95% CI: 22.1%–71.6%) and a 20-year risk of 91.2% (95% CI: 50.5%–98.4%). The 20-year risk decreased to 59.9% (95% CI: 21.9%–79.5%) if only one risk factor was met and to 35.7% (95% CI: 0.0%–66.2%) if the relative was older than 20 years with one autoantibody.

**Conclusions:**

In first-degree relatives with autoimmunity, the time to progression to T1D is faster in children and adolescents with multiple autoantibodies. Young adults are also at risk, which supports their consideration in screening strategies for people at risk of developing T1D.

## 1 Introduction

Type 1 diabetes (T1D) is a chronic disease caused by progressive autoimmune destruction of the insulin-producing pancreatic β-cells, leading to lifelong insulin dependence (1). The disorder can be identified in early presymptomatic stages such as stage 1, which represents individuals who have developed pancreatic autoimmunity with normoglycemia, and stage 2, which includes individuals who have progressed at a variable rate toward dysglycemia but remain asymptomatic. Individuals who have become clinically symptomatic are considered stage 3 (2). To date, pancreatic autoantibodies are the best markers with potential to identify people at risk of developing T1D in early presymptomatic stages (3). From a clinical and research perspective, insulin autoantibodies (IAA), glutamate decarboxylase autoantibodies (GADA), islet tyrosine phosphatase 2 autoantibodies (IA2A), and zinc transporter-8 autoantibodies (ZnT8A) are the most predictive of the development of diabetes (1, 3).

Although the etiology of the disease is still unknown, it is assumed that the autoimmune process is triggered by complex interactions between several genetic loci (nearly 60 loci described so far) and environmental factors (4–6). The highly polymorphic region harboring the class II HLA genes on chromosome 6p21 has been proposed as the main genetic factor, contributing to 40%–50% of the genetic risk. In European ancestry populations, HLA-DRB1 haplotypes DRB1*03:01-DQA1*05:01-DQB1*02:01 and DRB1*04-DQA1*03:01-DQB1*03:02 provide the highest risk of developing diabetes, especially when both haplotypes are present in heterozygosis (7, 8).

T1D is typically considered a disease of childhood and adolescence, but it affects people of all ages. In fact, recent epidemiological data have shown that more than half of all new cases of type 1 diabetes occur in adults (9, 10). Identification of people at risk is increasingly relevant due to recent success in delaying clinical disease in presymptomatic individuals using immunotherapy (11). First-degree relatives of patients with T1D have an increased risk of disease occurrence (approximately 15–20 times higher than the general population) (12). Early prediction of the disease in this population could reduce complications at diagnosis and provide an opportunity to delay the onset of T1D too (12). Therefore, the main objective of this study was to assess pancreatic autoimmunity in first-degree relatives of patients with T1D, estimate the time from autoimmunity detection to clinical diabetes onset, and identify the associated risk factors. These data provide essential information to design screening strategies for people at risk of T1D.

## 2 Methods

### 2.1 Study design and participants

Participants included in the study were identified from a serum sample collection database resulting from more than 25 years of autoimmune diabetes research conducted by our laboratory. In detail, this is a retrospective review of a prospectively maintained database including new onset patients with diabetes and their family members, all recruited between January 1992 and December 2018 at six tertiary reference hospitals distributed all over Spain. A total of 3,015 first-degree relatives of patients of European ancestry diagnosed with T1D were selected for the study (2,050 progenitors, 965 siblings). The diagnosis of T1D was made according to the American Diabetes Association (ADA) criteria (13). Relatives were tested for pancreatic autoantibodies at the time of proband diagnosis. Until 2007, three autoantibodies were analyzed for the diagnosis of T1D (IAA, GADA, and IA2A), and from this date, ZnT8A was added. In first-degree relatives screened before 2007, if any of the three autoantibodies tested were positive or if they were of pediatric age, autoimmunity analysis was completed with ZnT8A using the serum sample stored in the collection.

Family members with positive autoimmunity and normal fasting blood glucose without clinical symptoms of diabetes were included in the study. They were grouped into two age categories according to age at autoimmunity detection: children and adolescents (<20 years) and adults (≥20 years). These relatives were examined in the hospital every 3–12 months according to the endocrinologist’s criteria to assess for the occurrence of diabetic symptoms. The check-up included fasting blood glucose and glycated hemoglobin A1c (HbA1c) measurements and a complete testing for all four autoantibodies of interest. Follow-up for this study ended at the time of diagnosis of the disease. In addition, 13 siblings who tested negative for autoimmunity in the first sample collection were also followed up. Baseline characteristics of the population under study are shown in **Table 1**.

**Table 1:** Baseline characteristics of first-degree relatives of patients with T1D included in the study. Comparison between siblings with and without autoantibodies during follow-up.

	Progenitors with Ab (+) n=21	Siblings with Ab (+) n=27	Siblings with Ab (-) n=13	p-value
**Sex (male)**	12/21 (57.1)	12/27 (44.4)	7/13 (53.8)	0.577 ^a^
**Age at study entry (years)**	43.7 (38.1-47.7)	7.6 (4.4-15.6)	8.1 (5.8-16.1)	0.479 ^c^
Number of Ab at entry				
1 positive Ab	16/21 (76.2)	10/27 (37.0)	0	_
2 or more positive Abs	5/21 (23.8)	17/27 (63.0)	0	_
Ab type at entry				
IAA	5/21 (23.8)	15/27 (55.6)	0	_
GADA	16/21 (76.2)	23/27 (85.2)	0	_
IA2A	3/21 (14.3)	7/27 (25.9)	0	_
ZnT8A	5/21 (23.8)	9/27 (33.3)	0	_
HLA-DRB1 genotype				
High risk	1/20 (5.0)	9/21 (42.9)	0/13 (0.0)	0.006 ^b^
Moderate risk	16/20 (80.0)	8/21 (38.1)	7/13 (53.8)	0.369 ^a^
Low risk	3/20 (15.0)	4/21 (19.0)	6/13 (46.2)	0.130 ^b^

Age is shown as median (IQR: P_25_–P_75_). Rest of variables are shown as n/total (%). Ab, autoantibody. High risk (DR3/DR4), moderate risk (DR3/DR3, DR4/DR4, DR3/DRX, DR4/DRX), and low risk (DRX/DRX). DRX corresponds to any HLA-DRB1 allele different from DR3 and DR4. The DRB1*0403 allele is considered DRX. The p-value corresponds to the comparison between siblings with and without autoantibodies during follow-up. ^a^Pearson’s chi-square test. ^b^Fisher exact test ^c^Mann–Whitney U-test.

"-" means not applicable.

Detailed clinical markers on metabolic decompensation at diagnosis were collected from medical records of the probands and first-degree relatives who progressed to T1D over the course of the study. Data included serum glucose and HbA1c at diagnosis, presence of diabetic ketoacidosis (DKA) according to ADA criteria (14), duration of hospital stay, and admission at the intensive care unit (ICU), if needed.

The research was carried out in accordance with the Declaration of Helsinki (2008) of the World Medical Association. The study was approved by our local ethics committee, CEIm-E (Ethics Committee for Research with Medical Products of Euskadi), and informed consent was obtained from all participants and/or their legal guardians.

### 2.2 Autoantibody analyses

Pancreatic autoantibodies (IAA, GADA, IA2A, and ZnT8A) were determined in serum by standardized radioimmunoassay, as previously described (15). In brief, IAA was determined using a competitive fluid-phase radioassay that uses [^125^I]-labeled recombinant human insulin (PerkinElmer Inc, Waltham, MA, USA) as antigen. GADA, IA2A, and ZnT8A were determined by means of standardized radiobinding assays using *in vitro* transcribed and translated, [^35^S]-labeled, recombinant human full-length glutamate decarboxylase, IA2ic (amino acids 605–979), and ZnT8 antigens. The ZnT8A assay simultaneously measures autoantibodies against both variants of the COOH-terminal domain (Arg325/Trp325). Our laboratory has participated in different pancreatic islet autoantibody standardization program workshops, the last one in 2023. Specificity was 100% for all four antibody assays, and sensitivity was 65% for IAA, 80% for GADA, 78% for IA2A, and 70% for ZnT8A.

In first-degree relatives, determinations of autoantibodies were initiated when the proband was diagnosed. An individual was considered to have developed autoimmunity if the result was positive in at least two consecutive blood samples during the follow-up. The occurrence of more than one positive autoantibody was denoted as multiple autoantibodies.

### 2.3 HLA-DRB1 genotyping

HLA-DRB1 class II typing was performed by the PCR sequence-specific oligonucleotide method combined with Luminex technology using LABType RSSOH2B1 (HLA-DRB1-HD) commercial kit (One Lambda, Inc, Canoga Park, CA, USA). All procedures were performed according to the manufacturer’s instructions. HLA-DRB1 risk alleles for autoimmune diabetes were defined based on our previous report (16). The HLA-DRB1 genotypes were classified as high-risk (DR3/DR4), moderate risk (DR3/DR3, DR4/DR4, DR3/DRX, DR4/DRX), and low risk (DRX/DRX). DRX corresponds to any HLA-DRB1 allele different from DR3 and DR4. The DRB1*0403 allele is also considered DRX.

### 2.4 Statistical analyses

Qualitative variables were described as percentages and non-parametric quantitative variables as median and interquartile range (IQR: P_25_–P_75_). For comparisons, chi-square or Fisher’s exact test for categorical data and Mann–Whitney U-test for continuous data were applied as appropriate.

The association of different clinically relevant variables with the time to progression to diabetes was analyzed by time-dependent Cox proportional-hazards regression models. The starting univariate analyses included sex, relationship with the proband, age at autoimmunity detection, number and type of positive autoantibody, and HLA-DRB1 genotype. The variables with univariate regression p-value < 0.1 were included in the multivariate regression analysis. At each step of the modeling process, the variable with the highest p-value was eliminated and the model was tested again until all remaining variables were significant. Results were expressed as hazard ratios (HR) and 95% confidence intervals (95% CI). The significance level was defined as p-value < 0.05. To assess the discrimination power of the model, the area under the receiver operating characteristic (AUC–ROC) curve and the 95% confidence interval (95% CI) was calculated. The goodness-of-fit model was assessed using a Nam–Hosmer–Lemeshow test.

The cumulative risk of progression to diabetes in first-degree relatives who met two, one, or none of the highest risk factors according to the Cox proportional-hazards multivariate model (multiple autoantibodies and early age at autoimmunity detection), was estimated using Kaplan–Meier survival analysis, and the log-rank test was used to compare estimates. All statistical analyses were carried out using SPSS software (v.29; SPSS Inc., Chicago, IL).

## 3 Results

### 3.1 First-degree relatives with positive pancreatic autoimmunity

**Figure 1:**
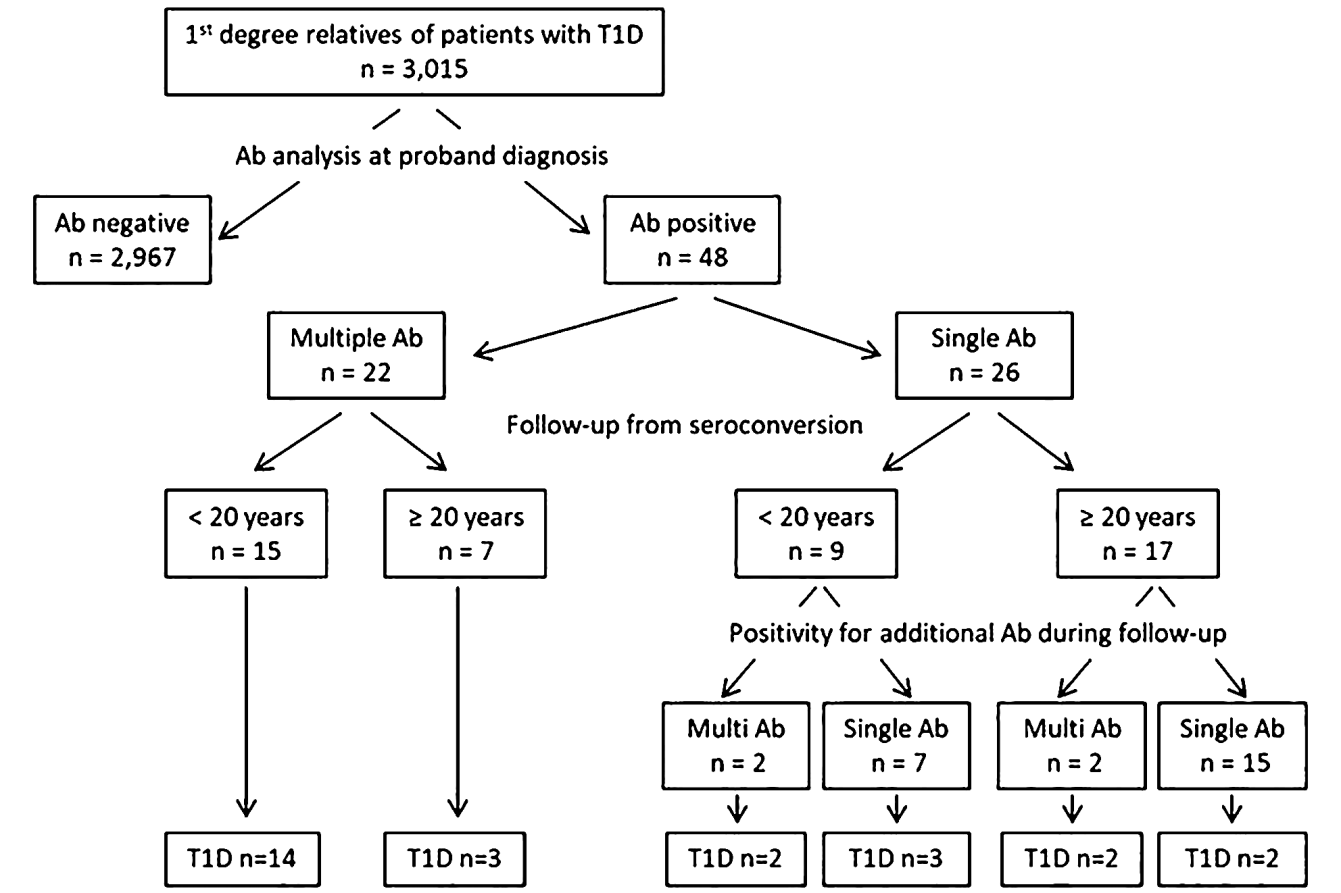
First-degree relatives who developed diabetes during follow-up according to the age at study entry and the number of positive autoantibodies. Age < 20 years are considered children and adolescents and age ≥ 20 years are considered adults. T1D, type 1 diabetes. Ab, autoantibody.

As illustrated in **Figure 1**, among the 3,015 first-degree relatives of patients with T1D [38.9 years (IQR: 24.9–44.9); 49.1% male] who were tested for autoimmunity at the time of proband diagnosis, 48 were positive for at least one of the four autoantibodies tested. These autoantibody-positive relatives were from 45 unrelated families and comprised 21 progenitors [43.7 years (IQR: 38.1–47.7); 57% male] and 27 siblings [7.6 years (IQR: 4.4–15.6); 48% male]. All families had a proband diagnosed with T1D at pediatric age [9.3 years (IQR: 5.2–12.2); 46.7% male], and a median of three family members per proband were tested (IQR: 2–4; ranged from 1 to 6). At initial sampling, GADA was the most prevalent autoantibody in 81.3% of the autoimmune-positive relatives, followed by IAA, ZnT8A, and IA2A (41.7%, 29.2%, and 20.8%, respectively). Of the 26 relatives with a single positive autoantibody at study entry, four developed positivity for additional autoantibodies over the course of follow-up.

In total, 54.2% (95% CI: 39.2%–68.6%) of the first-degree relatives with positive autoimmunity developed diabetes in a median of 5 years (IQR: 3.6–8.7; ranged from 0.9 to 22.6 years). The age at autoimmunity detection of these relatives ranged from 11 months to 39.4 years. The differences in the time to progression to T1D between those younger and older than 20 years were not statistically significant: 4.8 years (IQR: 3.0–7.2) vs. 7.6 years (IQR: 4.9–12.6), respectively (p = 0.107). The follow-up time of the remaining family members with autoimmunity and without symptoms of diabetes was 6.8 years (IQR: 3.7–24.1).

The frequency of the high-risk HLA-DRB1 genotype (DR3/DR4) was shown to be significantly higher among autoimmune-positive siblings compared with siblings who did not develop autoimmunity throughout the study (43% vs. 0%, p < 0.01). No differences were found with the frequencies of moderate- and low-risk HLA-DRB1 genotypes (**Table 1**).

Comparison of metabolic decompensation at diagnosis between probands and first-degree relatives who developed T1D during follow-up is shown in **Table 2**. Both probands and relatives had markedly elevated serum glucose levels, but HbA1c was lower in those relatives under health surveillance (p = 0.001). In addition, the percentage of patients with ketoacidosis at diagnosis was lower in first-degree relatives (p = 0.004); therefore, the follow-up of patients with positive autoimmunity resulted in significantly less severe symptoms of diabetes at diagnosis, requiring shorter hospitalization or none (p < 0.001).

**Table 2:** Comparison of metabolic decompensation at diagnosis of T1D between probands and first-degree relatives who developed T1D during follow-up.

	Probands n=22	First-degree relativesn=23	p-value
**Age at diagnosis (years)**	8.5 (5.2-11.4)	16.1 (9.9-26.5)	0.003^c^
**Sex (male)**	11/22 (50.0)	10/23 (43.5)	0.661^a^
**Glucose (mmol/L)**	18.6 (13.3-29.9)	16.4 (12.2-21.7)	0.154^c^
**HbA1c (%)**	10.0 (8.2-11.3)	7.4 (6.7-9.0)	0.001^c^
**Presence of DKA**	9/22 (40.9)	1/23 (4.3)	0.004^b^
**Hospital stay (days)**	8.5 (6.7-11.0)	0.0 (0.0-4.0)	< 0.001^c^
**ICU admission (days)**	5/22 (22.7)	0/23 (0.0)	0.022^b^

Sex, DKA, and ICU admission are shown as n/total (%). Rest of variables are shown as median (IQR: P_25_–P_75_). DKA, diabetic ketoacidosis. ICU, intensive care unit. HbA1c, glycated hemoglobin A1c. ^a^Pearson’s chi-square test. ^b^Fisher exact test. ^c^ Mann–Whitney U-test.

### 3.2 Risk factors associated with the time to diabetes development

The effect of a set of clinically relevant variables on the time to develop diabetes after the detection of pancreatic autoimmunity is shown in **Table 3**. The starting univariate analyses showed that among the 26 first-degree relatives who progressed to diabetes, 81% were siblings of the proband (HR = 3.06; 95% CI: 1.15–8.12; p = 0.025), 73.1% developed autoimmunity before the age of 20 years (HR = 3.04; 95% CI: 1.28–7.25; p = 0.012), and 81% had multiple autoantibodies (HR = 4.14; 95% CI: 1.55–11.01; p = 0.004). No significant associations were found with sex, the type of positive autoantibody, or the HLA-DRB1 profile. The results of the subsequent multivariate analysis showed that progression to diabetes was faster in those relatives under 20 years of age with two or more positive autoantibodies (AUC-ROC = 0.73; 95% CI: 0.65–0.82; p = 0.813, Nam–Hosmer–Lemeshow test).

**Table 3:** Cox proportional hazard model for prediction of progression to diabetes in first-degree relatives of patients with T1D.

	Univariate		Multivariate	
	Hazard ratio (95% CI)	p-value	Hazard ratio (95% CI)	p-value
**Sex (ref men)**	1.43 (0.66-3.08)	0.369	−	−
**Sibling of the proband (ref progenitor)**	3.06 (1.15-8.12)	0.025	ns	ns
**< 20 years at Abs detection (ref ≥ 20 years)**	3.04 (1.28-7.25)	0.012	2.58 (1.07-6.19)	0.034
**Multiple Abs in the study (ref single Ab)**	4.14 (1.56-11.01)	0.004	3.63 (1.35-9.74)	0.010
Ab type (ref Ab absent)				
IAA	1.13 (0.52-2.45)	0.753	−	−
GADA	1.77 (0.61-5.16)	0.294	−	−
IA2A	1.86 (0.80-4.33)	0.150	−	−
ZnT8A	1.18 (0.53-2.65)	0.691	−	−
HLA_DRB1 genotype (ref low risk)				
Moderate risk	0.67 (0.20-2.19)	0.510	−	−
High risk	1.11 (0.32-3.81)	0.866	−	−

Ab, autoantibody. High risk (DR3/DR4), moderate risk (DR3/DR3, DR4/DR4, DR3/DRX, DR4/DRX), low risk (DRX/DRX). DRX corresponds to any HLA-DRB1 allele different from DR3 and DR4. DRB1*0403 allele is considered DRX. Hazard ratios (95% CI) were calculated for each variable by univariate or multivariate Cox proportional hazard regression analysis, as indicated, with follow-up time as dependent variable. ns, not significant. ref, reference value."-" means not applicable.

### 3.3 Progression rate according to age at autoimmunity detection and autoimmune profile

**Figure 2:**
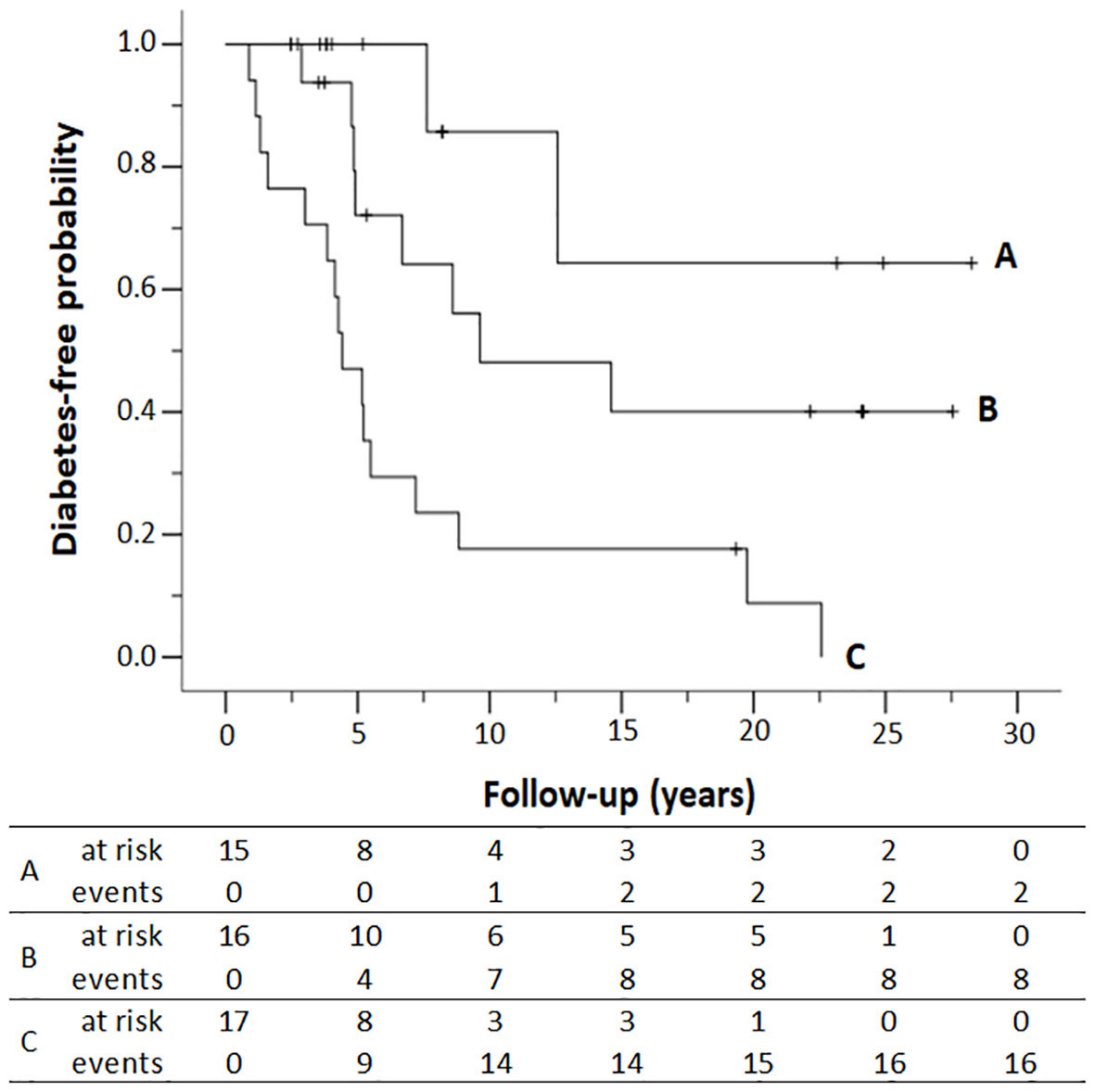
Diabetes-free survival of first-degree relatives according to the number of antibodies (Ab) detected in the study and the age at autoimmunity detection. **(A)** Single Ab and ≥ 20 years; **(B)** single Ab and < 20 years or multiple Ab and ≥ 20 years; **(C)** multiple Ab and < 20 years. Numbers at the bottom indicate the number of patients at risk of developing T1D and the number of patients who progressed to T1D (events) at each follow-up year. Kaplan–Meier lifetime analysis (p < 0.001, log-rank test).

The cumulative risk of progression to diabetes in first-degree relatives who met two, one, or none of the risk factors according to the Cox proportional-hazards multivariate model (early age at autoimmunity detection and multiple autoantibodies) is represented in **Figure 2**. Relatives with two or more positive autoantibodies and age less than 20 years showed a cumulative risk of developing diabetes of 52.9% (95% CI: 22.1%–71.6%) at 5 years after autoimmunity detection and increased to 82.3% (95% CI: 50.7%–93.7%) after 10 years. In those who met only one risk factor, i.e., single positive autoantibody and <20 years, or multiple positive autoantibodies and ≥20 years, the cumulative risk reaches 51.9% (95% CI: 15.5%–72.5%) after 10 years. The cumulative risk of diabetes after 20 years of follow-up was 91.2% (95% CI: 50.5%–98.4%) if both risk factors were met and decreased to 59.9% (95% CI: 21.9%–79.5%) if only one risk factor was met and to 35.7% (95% CI: 0.0%–66.2%) if the relative met none, i.e., was older than 20 years with a single autoantibody (p < 0.001, log-rank test).

## 4 Discussion

It is well-established that the detection of multiple pancreatic islet autoantibodies marks a preclinical stage of type 1 diabetes (2, 12). However, studies addressing the time to progression to diabetes after the detection of autoimmunity or the associated risk factors are limited and focus mainly on childhood and adolescence (17–19). This study includes first-degree relatives with autoimmunity regardless of age and provides data on progression to diabetes not only in childhood and adolescence but also in adults. Our data demonstrate that positivity for multiple autoantibodies together with an early age at autoimmunity detection are strong predictors of disease occurrence and accelerate the progression to T1D, similar to that observed in populations of European origin (8, 17, 18) and others (20). However, it should be noted that in half of the relatives with positive autoantibodies, the autoimmunity was detected in adulthood, and approximately one-third of them also progressed to diabetes. Therefore, young adults are also at risk of developing diabetes in a similar way to children and adolescents and should be included in the screening for autoimmune-positive relatives who could benefit from new therapies to prevent or delay the onset of the disease.

In contrast, no association with the time to progression to diabetes has been found with other recognized potential risk factors, such as HLA-DRB1 genotype. This result is consistent with different studies in which progression to diabetes in relatives after the onset of pancreatic autoimmunity was not influenced by the presence of the DR3/DR4 high-risk genotype (19, 21). One explanation for this lack of association can be found in the selection criteria of the study population based on the presence of autoimmunity. It is well documented that the HLA-DRB1 genotype plays an important role in the development of autoimmunity (22), and consequently, the HLA-DRB1 risk alleles in our cohort are highly represented. This fact, together with the sample size, could influence the statistical result. However, this does not mean that HLA-DRB1 is irrelevant in the development of diabetes, as demonstrated in this study by the higher frequency of high-risk HLA-DRB1 genotypes in siblings who developed autoimmunity compared with those who did not.

Recent studies have focused on the early detection of pancreatic islet autoantibodies to prevent or delay the progression to diabetes. This approach highlights the importance of knowing the time from the onset of autoimmunity to clinical diabetes, which is susceptible to immunotherapeutic intervention. Our results show that first-degree relatives with multiple positive autoantibodies and age less than 20 years at autoimmunity detection had a 5-year risk of developing diabetes of 52.9% and a 20-year risk of 91.2%. This period of time could be underestimated due to the imprecise time of autoimmunity onset in the relative, as the follow-up started at the diagnosis of the proband. Nevertheless, our findings are consistent with the resulting times from previous studies (17, 21, 23, 24). It is worth noting that GADA, the most prevalent autoantibody in adults, can also be found in other autoimmune disorders which can lead to false-positive results, especially in adults. Overall, the false-positive rate in autoantibodies testing is estimated at 1%–7% (10). Therefore, in our cohort, the possibility of false-positive autoimmunity among the 15 adult first-degree relatives with a single positive autoantibody cannot be ruled out. Nevertheless, this percentage is small and would not be expected significantly influence the results.

On the other hand, the time to progression for each individual varies considerably in our study, as previously published (25, 26). A special case is one sibling with high-risk HLA-DRB1 genotype and four positive autoantibodies at the age of 6 years who remains non-diabetic for more than 19 years. Although such situations are isolated cases because positivity for two or more autoantibodies seems to reflect irreversible progression to diabetes, it is essential to study factors associated with slower progression as it might be useful in understanding natural protective mechanisms. It has been suggested that genotypes of non-HLA genes could influence the duration of the latent phase of diabetes among children with multiple autoimmunity (19, 27) or that undetermined immunological and environmental risk factors could also be relevant to the progression (8).

In our cohort, it is remarkable that screening for autoimmunity in first-degree relatives has resulted in milder metabolic decompensation at diagnosis compared with patients diagnosed without follow-up (probands). This is in accordance with the reduction of ketoacidosis at the onset of diabetes reported by screening for pancreatic autoantibodies in the general population (28) or in newly diagnosed children with family history of diabetes (29). In addition, recent successful results with teplizumab (anti-CD3) and other underway therapies have opened opportunities for prevention or delayed the clinical diagnosis of T1D (11, 12, 25) and underlines the importance of identifying high-risk individuals who may benefit from these emerging treatments. Unfortunately, in this cohort, it was not possible to intervene on at-risk relatives due to the retrospective study design. Nevertheless, the results are essential to be taken into account in the screening strategies that should be implemented due to the upcoming availability of new advances in diabetes prevention (12).

A considerable strength of the study is the multicenter design conducted with a relatively large number of participants over a remarkably long period of time. However, a large cohort of first-degree relatives with positive autoimmunity prior to clinical symptoms has not been achieved due to the low prevalence of the disease, but it is precisely this difficulty in recruiting people with preclinical T1D that makes the sample, although small, of great value. In addition, despite the limitations discussed above, such as the possible inaccuracy in the timing of onset of autoimmunity in relatives and the retrospective design, our results are of potential interest to researchers and clinicians alike.

In summary, this study shows that the strongest predictors for progression to diabetes among first-degree relatives of patients with T1D include early age at autoimmunity detection and multiple positive autoantibodies. However, the time of progression for each individual can vary considerably and, although progression to diabetes is faster in childhood and adolescence, young adults are also at risk of developing the disease in a similar way. Accurate screening of individuals at risk is crucial to select candidates that could benefit from new therapies to prevent or delay the disease.

## Data Availability

The original contributions presented in the study are included in the article/Supplementary Material. Further inquiries can be directed to the corresponding authors.

## The collaborative working group

A. Aguayo, AC Aranaga-Decori, J. Aurrekoetxea-Oribe, C. Baquero, J. Corcuera, A. Cortázar, E. Fernández-Rubio, S. Gómez-Conde, G. Grau, S. Huerga, M. Sánchez-Morán, N. Valdés, A. Vela, O. Velasco (H.U. Cruces, Bizkaia, Spain); ML. Bertholt, C. Luzuriaga, C. Montalbán (H.U. Marqués de Valdecilla, Cantabria, Spain); G. Lou-Francés (C.S. Binefar, Huesca, Spain); B. García-Cuartero (H.U. Ramón y Cajal, Madrid, Spain); M. Chueca (H.U. de Navarra, Spain); M. Ferrer (H.U. Miguel Servet, Zaragoza, Spain); N. Pacho (H. Zumarraga, Gipuzkoa, Spain).
